# Trapping a Knot into Tight Conformations by Intra-Chain Repulsions

**DOI:** 10.3390/polym9020057

**Published:** 2017-02-10

**Authors:** Liang Dai, Patrick S. Doyle

**Affiliations:** 1 BioSystems and Micromechanics IRG, Singapore-MIT Alliance for Research and Technology Centre, Singapore 117543, Singapore; dailiang@smart.mit.edu; 2 Department of Chemical Engineering, Massachusetts Institute of Technology (MIT), Cambridge, MA 02139, USA

**Keywords:** knot, polymer, diffusion, Langevin dynamics simulation

## Abstract

Knots can occur in biopolymers such as DNA and peptides. In our previous study, we systematically investigated the effects of intra-chain interactions on knots and found that long-range repulsions can surprisingly tighten knots. Here, we use this knowledge to trap a knot into tight conformations in Langevin dynamics simulations. By trapping, we mean that the free energy landscape with respect to the knot size exhibits a potential well around a small knot size in the presence of long-range repulsions, and this potential can well lead to long-lived tight knots when its depth is comparable to or larger than thermal energy. We tune the strength of intra-chain repulsion such that a knot is weakly trapped. Driven by thermal fluctuations, the knot can escape from the trap and is then re-trapped. We find that the knot switches between tight and loose conformations—referred to as “knot breathing”. We use a Yukawa potential to model screened electrostatic interactions to explore the relevance of knot trapping and breathing in charged biopolymers. We determine the minimal screened length and the minimal strength of repulsion for knot trapping. We find that Coulomb-induced knot trapping is possible to occur in single-stranded DNA and peptides for normal ionic strengths.

## 1. Introduction

Knotting occurs in biopolymers, such as DNA [1,2,3,4,5] and proteins [6,7,8,9,10], and other polymers. Simulations have been performed to investigate knot behaviors under various conditions, such as in free space [1,11], in spatial confinement [12,13,14,15], under pulling forces [16,17], in good/bad solvents [18], in a crowding environment [19], with different bending stiffness [20,21], and during translocation through a nanopore [22,23]. Theory and simulations revealed that a metastable tight knot exists in semiflexible [24,25] and flexible chains [26] due to the self-tightening of knots by entropy [27]. In experiments, knots in DNA or filaments can be tied manually [28,29], formed spontaneously [30] or formed by compression [31,32]. The spontaneously-formed knot in fluorescently labeled DNA under tension [29] or in nanochannels [30] can be identified as a bright spot diffusing along DNA and disappearing only at one end. In gel electrophoresis, DNA molecules with different topologies migrate with different speeds [1,3,4,5]. The knots in DNA can also be identified by atomic-force microscopy (AFM) imaging [33]. Recently, DNA knots were identified by nanopore translocation experiments [34].

Recently, our group investigated the general effects of intra-chain interactions on knots, and accordingly explored how to control the knotting probability and the knot size by intra-chain interactions [35]. The physical origins of intra-chain interactions can be electrostatic interactions, depletion attractions in a crowding environment, van der Waals forces, or others. By using simple interaction forms for generality, we found that attractions (repulsions) usually increase (decrease) the knotting probability. However, long-range and short-long interactions have opposite effects on the knot size. Short-range repulsion tends to swell a knot, while long-range repulsion tends to tighten a knot. The reason is that a larger knot contains less pairs of monomers with short distances, but more pairs of monomers with long distances. Based on this knowledge, we can tighten a knot by long-range repulsion to any extent we want. Note that attractions cannot substantially tighten a knot, because strong attractions will lead to a coil–globule transition, and the knot core then spreads over the entire chain in a globular conformation [18]. In addition to controlling the knot size, the control of the knot position along a chain has been achieved through the inhomogeneity of bending rigidity along the chain, e.g., diblock flexible-stiff polymer [36]. The knot can be preferably located in the stiff region, the flexible region, or the interface of the two regions depending on the monomer–monomer interaction strength relative to the thermal energy, the sizes of flexible and stiff regions relative to the knot size, and the bending stiffness of the stiff region [36].

In this paper, we explore the phenomenon of trapping a knot in tight conformation via long-range repulsion. We purposefully tune the strength of repulsion such that a knot is moderately trapped in tight conformations and can escape from this trap by thermal fluctuations. From a time series of the escaping and then re-trapping of the knot, we can determine the lifetimes of the knot in tight conformations. In the first part of the result section, we will present the results of simulations using triangle potentials in order to make connection with our previous study as well as speed up simulations. In the second part of the result section, we will present the results for Yukawa potentials (screened Coulomb potentials), which represent screened electrostatic interactions. After determining the minimal screened length and the minimal strength of repulsion for knot trapping, we find that Coulomb-induced knot trapping can occur for single-stranded DNA and peptides under reasonable ionic strength conditions.

## 2. Simulation Methods

**Figure 1:**
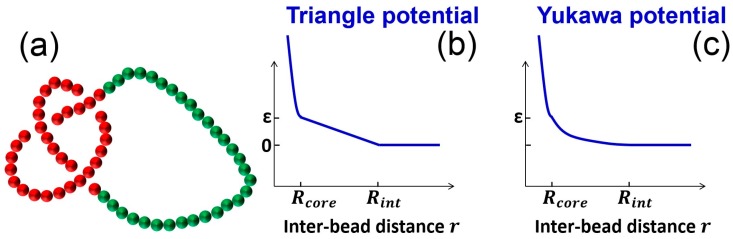
Simulation setup. (**a**) Snapshot of a knot in a homogenous circular chain. The monomers in the knot core are in red, while other monomers are in green; (**b**) Triangle potential for the pairwise interactions between monomers; (**c**) Yukawa potential (screened electrostatic interaction) for the pairwise interactions between monomers.

Langevin dynamics simulations are performed for ring chains using the LAMMPS program [37]. The circular chain is modeled by a bead-spring model [38] with an extra pairwise soft potential (Figure 1). The total pairwise interaction between monomers is a hardcore repulsion plus a soft potential, $E_{\mathrm{pair}}=E_{\mathrm{hard}}+E_{\mathrm{soft}}$. The hardcore pairwise interaction between monomers is described by a purely repulsive Lennard–Jones potential

(1) $$E_{\mathrm{hard}}=4\epsilon_{\mathrm{LJ}}\left[{\left(\frac{\sigma }{r}\right)}^{12}-{\left(\frac{\sigma }{r}\right)}^{6}\right]\;\mathrm{for}\;r\leq R_{\mathrm{core}}\equiv 2^{\frac{1}{6}}\sigma$$

with $\epsilon_{\mathrm{LJ}}=30\;k_{B}T$. The cutoff of Lennard–Jones potential is set as $R_{\mathrm{core}}\equiv 2^{\frac{1}{6}}\sigma \approx 1.1224\sigma$ to produce a purely repulsive potential. The hardcore diameter of the monomer can be approximated as σ. The soft pairwise interaction is either a triangle potential or Yukawa potential. The triangle potential takes the form:

(2) $$E_{\mathrm{triangle}}=\epsilon \left(R_{\mathrm{int}}-r\right)/R_{\mathrm{int}}\;\mathrm{for}\;r\leq R_{\mathrm{int}}$$

with an interaction range of $R_{\mathrm{int}}$ and an interaction strength of ϵ. The Yukawa potential takes the form:

(3) $$E_{\mathrm{Yukawa}}=\epsilon \exp\left(-\kappa r\right)/r$$

with a Debye length of $\kappa^{-1}$ and an interaction strength of ϵ. For practical reasons, a cutoff is needed for this potential and we set the cutoff as $10\kappa^{-1}$. The bond interactions between adjacent monomers are described by a FENE potential:

(4) $$E=-0.5KR_{0}^{2}\ln\left[1-{\left(\frac{r}{R_{0}}\right)}^{2}\right]+4\epsilon_{\mathrm{bond}}\left[{\left(\frac{\sigma }{r}\right)}^{12}-{\left(\frac{\sigma }{r}\right)}^{6}\right]+\epsilon_{\mathrm{bond}}$$

where the stiffness of the bond is set to $K=30\;k_{B}T/\sigma ²$, the maximum stretching distance $R_{0}$ is set as 1.5σ, and the hardcore repulsion between a pair of bonded monomers has a strength of $\epsilon_{\mathrm{bond}}=1.0\;k_{B}T$. Note that the normal pairwise repulsion described by Equation (1) is ignored for a pair of bonded monomers. For semiflexible chains, the bending energy is applied for every three adjacent monomers to reproduce a persistence length $L_{p}$:

(5) $$E_{\mathrm{bend}}=\left(1/2\right)\left(L_{p}/\sigma \right)\theta^{2}\;\left(k_{B}T\right)$$

where θ is the bending angle. A similar simulation model has been used by Matthews et al. [21] for knots. Recall that $L_{p}$ used in the current study corresponds to the intrinsic persistence length caused on the bending energy, while the apparent persistence length for the entire polymer conformation often deviates from this intrinsic persistence length due to interactions other than bending energy.

We normalize simulation times by the relaxation time of a single monomer $\tau_{\mathrm{bead}}=\sigma^{2}/D_{\mathrm{bead}}$, where $D_{\mathrm{bead}}$ is the diffusion coefficient of a single monomer. The diffusion coefficient $D_{bead}$ is calculated as $D_{\mathrm{bead}}=2k_{B}T\alpha /m$ with α as the damping time in Langevin dynamics simulation and m is the mass of a monomer. We set the time step as 0.02$\tau_{\mathrm{bead}}$. The time step is small enough to prevent two segments from crossing each other in one time step, and the topology is hence preserved during simulations. Figure 1 shows a chain conformation containing a trefoil knot, as well as pairwise interaction potentials. In most simulations, we run for $2\;\times \;10^{9}$ steps and save conformations every $10^{5}$ steps for analysis. In the simulations with slow knot dynamics, we run $10^{10}$ steps.

To determine the knot core, we cut monomers one by one from each end of the chain until the topology is changed. The topology is calculated by the Alexander polynomial, as done in our previous studies [25,39]. The number of beads in the knot core is defined as the knot size $L_{\mathrm{knot}}$. We approximate the contour length in the knot core as $L_{\mathrm{knot}}\sigma$, ignoring the fluctuation of bond length between two adjacent monomers. To determine the knot core for a circular chain, we need to break the circular chain at a point to form a linear chain. The breaking point at simulation step i is chosen based on the knot position at simulation step (i−1). Suppose that the chain at simulation step (i−1) has a knot at the position p, the breaking point at simulation step is at $p+N_{m}/2$, where $N_{m}$ is the number of monomers in the circular chain Such a method of choosing the breaking point is based on the fact that we save the conformation so frequently that the knot motion is small during every single step.

## 3. Results

### 3.1. Knot Breathing in a Flexible Chain with a Triangle Potential

**Figure 2:**
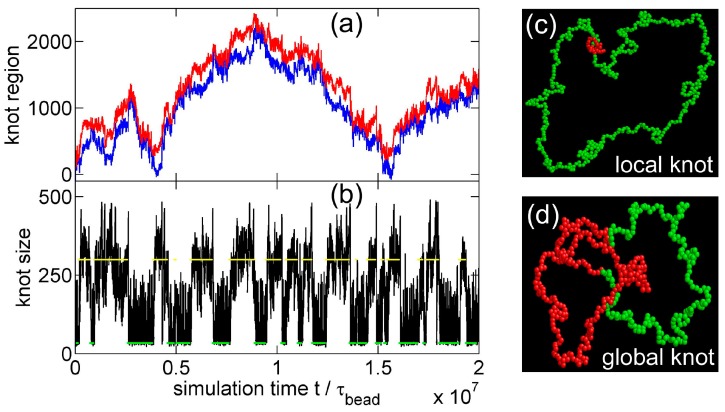
(**a**) The start (red line) and end (blue line) positions of a knot along a circular flexible chain in a simulation with the parameter set {A triangle potential, $N_{m}=500$, $R_{\mathrm{int}}$ = 10σ, ϵ=0.01 $k_{B}T$}. The positions are occasionally offset by $N_{m}=500$ to make both curves continuous. (**b**) The number of beads in the knot core as a function of the simulation time calculated from the top graph. The green and yellow lines indicate the most probable sizes of local and global knots, respectively. (**c**,**d**) are snapshots in this simulation, with the knot region denoted in red and the unknotted region denoted in green.

Figure 2 shows a typical simulation where a knot switches between tight conformations and loose conformations. We use a flexible chain with a triangle potential to speed up this simulation. For a flexible chain with a triangle repulsion, the critical interaction range for repulsion-induced knot tightening was determined as $R_{\mathrm{int}}^{*}\approx 6\sigma$ in our previous study [35]. Here, we set $R_{\mathrm{int}}=10\sigma$, which is much larger than $R_{\mathrm{int}}^{*}$. We set the strength of the triangle potential as $\epsilon =0.01\;k_{B}T$ such that the tight knot is moderately trapped.

**Figure 3:**
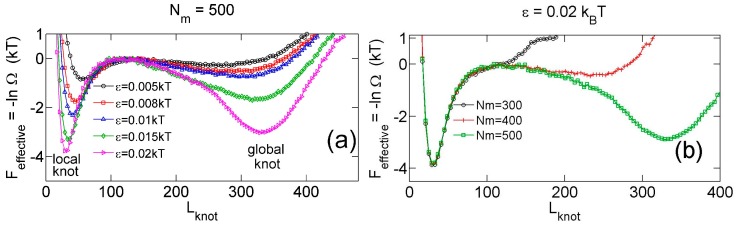
(**a**) Effective potential as a function of the knot size for various strengths of triangle potentials obtained from simulations with fixed $N_{m}=500$ and fixed $R_{\mathrm{int}}$ = 10σ. (**b**) Effective potential as a function of the knot size for various sizes of ring chains obtained from simulations with fixed $\epsilon =0.02\;k_{B}T$ and fixed $R_{\mathrm{int}}$ = 10σ.

Figure 2a,b show that the knot diffuses along the chain over a distance much larger than the knot size, and the knot size switches between two distinct states: a tight state with the most probable knot size $L_{\mathrm{knot}}^{*}\approx 41$, and a loose state. Recall that a flexible chain with pure hardcore repulsion has a metastable knot size of $L_{\mathrm{knot}}^{*}\approx 140$ [26]. The distribution of knot size is converted to effective potentials as shown in Figure 3. We highlight that the size of the tight state is insensitive to the entire chain size, while the size of the loose state increases with the chain size as shown in Figure 3 (right). As a result, we refer to the tight knots as local knots, and refer to the loose knots as global knots.

**Figure 4:**
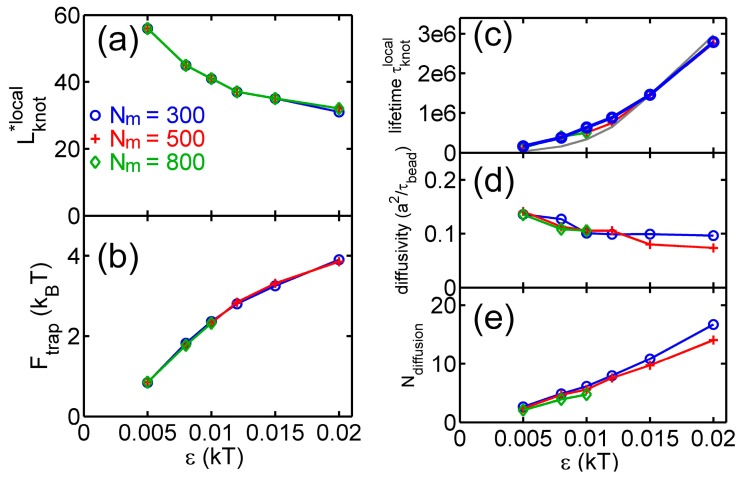
Results from simulations with parameter sets {a circular chain, $N_{m}$ = 300, 500 or 800, a triangle potential, $R_{\mathrm{int}}=10\sigma$}. (**a**) Metastable size of local knots. (**b**) The depth of potential well around local knots. (**c**) The lifetime $\tau_{\mathrm{knot}}^{\mathrm{local}}$ of local knots in units of $\tau_{\mathrm{bead}}$. The gray curve is calculated from Equation (7). The data for $N_{m}=800$ is incomplete at large ϵ due to an insufficient number of hopping events in simulations. (**d**) The diffusivity $D_{\mathrm{diffuse}}$ of a knot along the chain. The calculation of diffusivity is based on all data, including both tight and loose knots. (**e**) The normalized diffusion distance along the chain during the lifetime calculated from Equation (9).

Figure 4 shows various quantities about knots as a function of the strength of the triangle potential. As the repulsive triangle potential becomes stronger, the local knot becomes tighter, the effective trap becomes deeper, and the knot diffusion becomes slightly slower. The reduction of the diffusivity for the smaller knot was also observed in previous studies, because tighter knots experience larger intra-chain friction forces [17,40].

**Figure 5:**
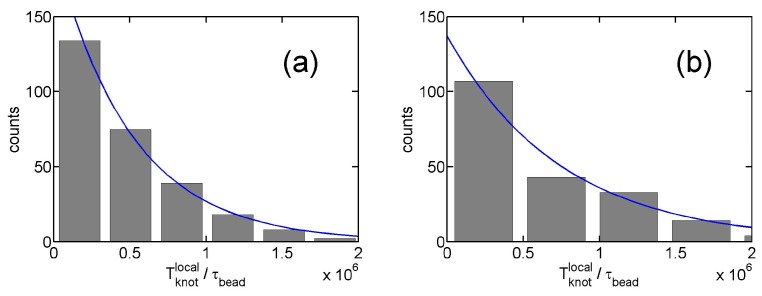
(**a**) Histogram of dwelling time of local knots in simulations. The simulation parameter set is {$N_{m}=500$, triangle potential, $R_{\mathrm{int}}=10\sigma$, $\epsilon =0.01\;k_{B}T$}. The solid line is the fit to an exponential function $counts=197\exp\left[T_{\mathrm{dwell}}/\left(5.0\times 10^{5}\tau_{\mathrm{bead}}\right)\right],$ from which we determine $\tau_{\mathrm{knot}}^{\mathrm{local}}\approx 5.0\times 10^{5}\tau_{\mathrm{bead}}$. (**b**) The result for $\epsilon =0.012\;k_{B}T$ and $\tau_{\mathrm{knot}}^{\mathrm{local}}\approx 7.5\times 10^{5}\tau_{bead}$.

From the stepwise evolution of knot size, we can calculate the dwelling time $T_{\mathrm{dwell}}$ of a knot trapped in the potential well around local or global knots. Figure 5 shows the histogram of $T_{\mathrm{dwell}}$. The distribution of dwelling time $T_{\mathrm{dwell}}$ appears to follow an exponential decay,

(6) $$P\left(T_{\mathrm{dwell}}\right)∼\exp\left(-T_{\mathrm{dwell}}/\tau_{\mathrm{knot}}^{\mathrm{local}}\right)$$

with a characteristic lifetime $\tau_{\mathrm{knot}}^{\mathrm{local}}$. An exponential distribution of dwelling time was also observed in a recent study of knots [40]. Figure 4c shows $\tau_{\mathrm{knot}}^{\mathrm{local}}$ increases with ϵ due to the increase of the trapping potential well. The lifetimes of local knots are insensitive to $N_{m}$. Based on a simple transition state theory, the lifetime can be approximated by the following equation:

(7) $$\tau_{\mathrm{knot}}^{\mathrm{local}}\approx \tau_{0}\exp\left(F_{\mathrm{trap}}\right)$$

where $\tau_{0}$ may be considered as the time scale for a knot to diffuse a certain distance in the absence of free energy barrier. We further make the following approximation:

(8) $$\tau_{0}\approx {\left(L_{\mathrm{knot}}^{\mathrm{barrier}}\sigma -L_{\mathrm{knot}}^{*}\sigma \right)}^{2}/D_{\mathrm{diffuse}}$$

where $L_{\mathrm{knot}}^{\mathrm{barrier}}\approx$100 is the knot size at the barrier as shown in Figure 3. The above equation assumes the speed of $L_{\mathrm{knot}}$ changing equals the diffusivity of a knot along the chain. The lifetime estimated by this approximation is in fair agreement with simulation results (the gray curve in Figure 4c).

Figure 4e shows the average distance of a local knot along the chain during the lifetime normalized by $L_{\mathrm{knot}}^{*}$:

(9) $$N_{\mathrm{diffuse}}\equiv \sqrt{D_{\mathrm{diffuse}}\tau }/\left(L_{\mathrm{knot}}^{*}\sigma \right)$$

The normalized diffusion distance $N_{\mathrm{diffuse}}$ monotonically increases with ϵ. It is worth noting that the lifetimes of knots are typically on the order of $10^{6}\tau_{\mathrm{bead}}$, which are much longer than the relaxation time ($∼10^{4}\tau_{\mathrm{bead}}$) of a unknotted chain with the same $N_{m}$. Such long lifetimes should provide convenient opportunities for experimental observation of knot breathing, i.e., switching between local and global knots.

### 3.2. Knot Breathing in a Semiflexible Chain with a Yukawa Potential

**Figure 6:**
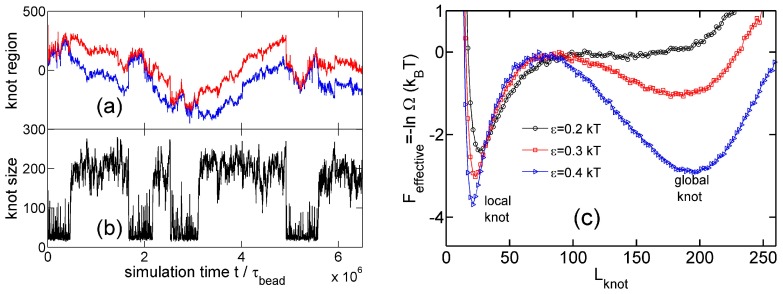
(**a**) The start (red line) and end (blue line) positions of a knot along a circular chain in a simulation {Yukawa potential, $N_{m}$ = 300, $L_{p}$ = 2.5σ, $\kappa^{-1}$ = 5σ, ϵ = 0.4 $k_{B}T$}. The positions are occasionally offset by $N_{m}$ = 300 to make both curves continuous. (**b**) The number of beads in the knot core calculated from the plot in (**a**). (**c**) Effective potentials as a function of the knot size for three strengths of Yukawa potentials. We fix $N_{m}$ = 300, $L_{p}$ = 2.5σ and $\kappa^{-1}$ = 5σ.

**Figure 7:**
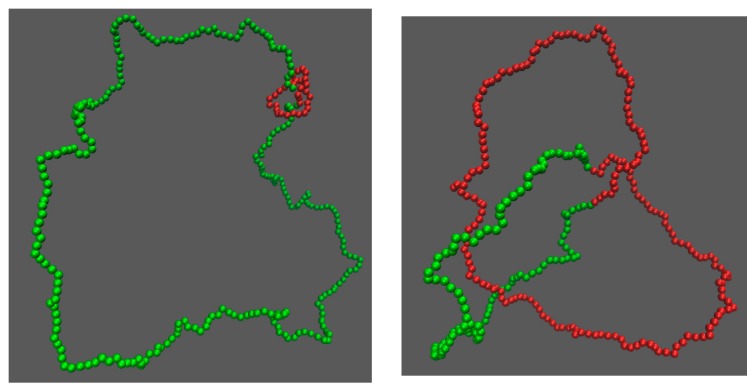
Simulation snapshots of the local knot (**left**) and the global knot (**right**) from the simulation {Yukawa potential, $N_{m}\;$= 300, $L_{p}$ = 2.5σ, $\kappa^{-1}\;$= 5σ, ϵ = 0.4 $k_{B}T$}. The red beads correspond to knot cores, while the green beads correspond to unknotted region.

Now we study knot breathing in a semiflexible chain with a Yukawa potential. Figure 6a,b shows an example of the diffusion of a trefoil knot along a circular chain. We use a weak bending stiffness $L_{p}=2.5\sigma$, because a larger $L_{p}$ requires a chain with more monomers to eliminate the finite-length effects and accordingly requires more computational efforts. We use a Yukawa repulsion with $\kappa^{-1}=5\sigma$ and ϵ = 0.4 $k_{B}T$ to moderately squeeze the knot size to $L_{\mathrm{knot}}^{*}\approx 21$. Simulation snapshots of tight and loose knot conformations are presented in Figure 7. From the distribution of the knot size, we calculate the effective potential as a function of the knot size as shown in Figure 6c. The traps around local and global knots becomes deeper as the Yukawa potential becomes stronger.

**Figure 8:**
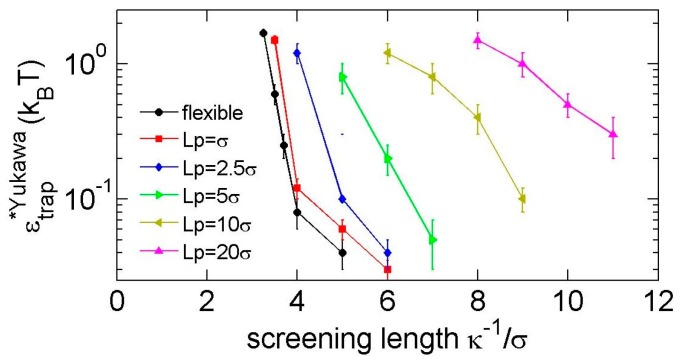
The minimal strengths of Yukawa repulsions for trapping a trefoil knot.

To substantially trap a knot, a minimum strength $\epsilon_{\mathrm{trap}}^{*}$ is required. Figure 8 shows the minimal strengths for Yukawa repulsions. We consider a knot to be substantially trapped if the life time of the local knots is more than 10 times the relaxation time of a local knot in simulations. The leftmost point of each curve in Figure 8 roughly indicates the minimal screening length $\kappa^{-1*}$ to trap a knot. Note that repulsion-induced knot trapping is a phenomenon requiring a criteria stricter than repulsion-induced knot shrinking in our previous study [35]. For repulsion-induced knot shrinking, we treat the soft potential as a weak perturbation and judge the trend of $\partial L_{\mathrm{knot}}^{*}/\partial \epsilon$ around ϵ=0. Recall that $\partial L_{\mathrm{knot}}^{*}/\partial \epsilon$ < 0 corresponds to the repulsion-induced knot shrinking, while $\partial L_{\mathrm{knot}}^{*}/\partial \epsilon$ > 0 corresponds to the repulsion-induced knot swelling. Sometimes, $\partial L_{\mathrm{knot}}^{*}/\partial \epsilon$ will switch from a negative value to a positive value as we increase ϵ from zero to a finite positive value. Under that situation, we cannot trap a knot by repulsion. For repulsion-induced knot trapping, we use a finite repulsion to substantially trap a knot. Both repulsion-induced knot shrinking and repulsion-induced knot trapping require a sufficiently long-range interaction. In the case of flexible chains, the minimal screening length for repulsion-induced knot shrinking is $\kappa_{\mathrm{shrink}}^{-1}\approx 1.6\sigma$, while the minimal screening length for repulsion-induced knot trapping shrinking is $\kappa_{\mathrm{trap}}^{-1}\approx 3.3\sigma$. It means that, in the case of $1.6\sigma <\kappa_{\mathrm{shrink}}^{-1}<3.3\sigma$, the knot will shrink and then swell as we increase the strength of repulsion from ϵ=0, and we cannot substantially trap a knot.

**Figure 9:**
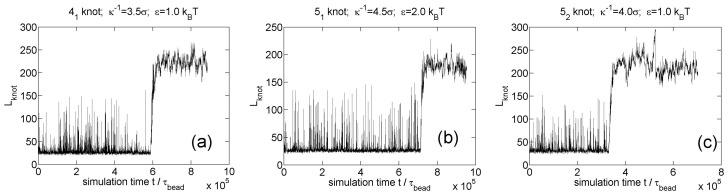
Knot breathing for other knot types in simulations of circular flexible chains with 300 monomers and Yukawa potentials. (**a**) $4_{1}$ knot; (**b**) $5_{1}$ knot; (**c**) $5_{2}$ knot.

We also simulate knot breathing for other knot types ($4_{1}$, $5_{1}$, $5_{2}$ knots) as shown in Figure 9. The three screening lengths in these three simulations are close to the minimal screening lengths for knot trapping. The minimal screening length does not appear to increase with the complexity of knot, which was also found in our previous study [35].

## 4. Discussion about Length Scales and the Relevance to DNA and Peptides

Using the monomer size of the length unit, there are three characteristic length scales in our simulation system: the knot size $L_{\mathrm{knot}}$, the persistence length $L_{p}$ and the range of soft interaction $R_{\mathrm{int}}$. The interplay among these three length scales are quite complicated. Our previous study [35] indicated that the combination of the persistence length and the knot size leads to a pair correlation between monomers, which has a characteristic length corresponding to the critical interaction range $R_{\mathrm{int}}$. While we pointed out the central role of the pair correlation, we have not yet arrived at a simple physical picture to describe the interplay between $L_{\mathrm{knot}}$, $L_{p}$, and $R_{\mathrm{int}}$. Even in the absence of soft interaction, the interplay between $L_{\mathrm{knot}}$ and $L_{p}$ can lead to an intriguing phenomenon. Matthews et al. found that the free energy cost of knot formation on a chain under tension is minimized at a non-zero critical value of persistence length $L_{p}^{*}$, while $L_{p}^{*}$ depends on the knot size, which is controlled by a pulling force [21].

It is interesting to examine whether the Coulomb-induced knot trapping can be applied to biopolymers, such as DNA and peptides. In order to induce the knot trapping, the interaction range of electrostatic interaction needs to be larger than a critical value, which means the ionic strength needs to be smaller than a critical value. We estimate the ionic strengths required to induce the Coulomb-induced knot trapping in DNA and peptides. For double-stranded (ds) DNA, we approximate σ≈2.5 nm and $L_{p}\approx 50$ nm. We obtain the minimal screening length is $\kappa^{-1}\approx 20$ nm. Applying $\kappa^{-1}\approx 0.304\;\mathrm{nm}/\sqrt{I\left(\mathrm{in}\;\mathrm{mol}/L\right)}$, we estimate that the ionic strength I should satisfy I<0.23 mM for Coulomb-induced knot trapping. For single-stranded (ss) DNA, we approximate σ≈1 nm and $L_{p}\approx 3\;$nm [41]. We have the minimal screening length $\kappa^{-1}\approx 4$ nm, corresponding to the ionic strength of 5.8 mM. When considering peptides as flexible chains with σ≈0.36 nm, we estimate the minimal screening length $\kappa^{-1}\approx 1.17$ nm, corresponding to the ionic strength of 68 mM. These estimations suggest that Coulomb-induced knot trapping can occur for ss-DNA and peptides with a few millimolar ionic strengths, and is not likely for ds-DNA unless the ionic strength is sub-mM. Physiological conditions have an ionic strength of about 150 mM, which is probably too high to induce the Coulomb-induced knot trapping.

Next, we estimate the prefactor ϵ in Yukawa potential for ss-DNA and peptides. The prefactor ϵ can be considered as the Coulomb interaction energy between two charges with separation σ in a medium of dielectric constant ≈80. For ss-DNA, the charge in each nucleic acid is −1e and the separation is σ≈1 nm, and we then obtain ϵ≈0.69 $k_{B}$T. In the case of peptides with amino acid separation of σ≈0.36 nm, if the amino acid has 1e or –1e, then we obtain ϵ≈1.92 $k_{B}$T. The critical ϵ in Figure 8 is usually less than 1 $k_{B}T$, and the strengths of Coulomb interactions in ss-DNA and peptides thus may be sufficient for knot trapping. In the case of double-stranded DNA, the charge density is −1e per 0.17 nm length, and a bead of size 2.5 nm carries a charge of about −14.7e. Accordingly, we obtain ≈59.8 $k_{B}T$. Once the Debye screening length is large enough for the Coulomb-induced knot trapping in double-stranded DNA, the interaction strength is not a problem.

It is worth mentioning that, in single-stranded DNA, the hydrophobic attraction between bases in two different nucleic acids should strongly affect the knot conformation and dynamics, and may overwhelm the Coulomb-induced knot trapping or hinder the knot reaching its equilibrium conformation.

## 5. Conclusions

In this work, we use long-range pairwise repulsions between monomers to trap knots in tight conformations. We tune the strength of repulsion so that a knot is moderately trapped in tight conformations, and we can then observe knot breathing, the escaping and re-trapping of the knot. We determine the minimal strengths of Yukawa potentials (screened electrostatic interaction) as well as the minimal screening lengths for knot trapping. We find that knot trapping can be induced by electrostatic interactions in single-stranded DNA and peptides under normal ionic strength. For double-stranded DNA, the Coulomb-induced knot trapping may occur under very low ionic strength with I<0.23 mM.

## References

[1] Rybenkov V.V., Cozzarelli N.R., Vologodskii A.V. (1993). Probability of DNA knotting and the effective diameter of the DNA double helix. Proc. Natl. Acad. Sci. USA.

[2] Rybenkov V.V., Ullsperger C., Vologodskii A.V., Cozzarelli N.R. (1997). Simplification of DNA topology below equilibrium values by type II topoisomerases. Science.

[3] Arsuaga J., Vazquez M., Trigueros S., Sumners D., Roca J. (2002). Knotting probability of DNA molecules confined in restricted volumes: DNA knotting in phage capsids. Proc. Natl. Acad. Sci. USA.

[4] Arsuaga J., Vazquez M., McGuirk P., Trigueros S., Sumners D., Roca J. (2005). DNA knots reveal a chiral organization of DNA in phage capsids. Proc Natl. Acad. Sci. USA.

[5] Marenduzzo D., Orlandini E., Stasiak A., Sumners D., Tubiana L., Micheletti C. (2009). DNA-DNA interactions in bacteriophage capsids are responsible for the observed DNA knotting. Proc Natl. Acad. Sci. USA.

[6] Taylor W.R. (2000). A deeply knotted protein structure and how it might fold. Nature.

[7] Virnau P., Mirny L.A., Kardar M. (2006). Intricate knots in proteins: Function and evolution. PLoS. Comput. Biol..

[8] Lai Y.L., Yen S.C., Yu S.H., Hwang J.K. (2007). pKNOT: The protein KNOT web server. Nucleic Acids Res..

[9] Dai L., Zhou Y. (2011). Characterizing the existing and potential structural space of proteins by large-scale multiple loop permutations. J. Mol. Biol..

[10] Ziegler F., Lim N.C., Mandal S.S., Pelz B., Ng W.P., Schlierf M., Jackson S.E., Rief M. (2016). Knotting and unknotting of a protein in single molecule experiments. Proc. Natl. Acad. Sci. USA.

[11] Orlandini E., Whittington S.G. (2007). Statistical topology of closed curves: Some applications in polymer physics. Rev. Mod. Phys..

[12] Micheletti C., Marenduzzo D., Orlandini E. (2011). Polymers with spatial or topological constraints: Theoretical and computational results. Phys. Rep..

[13] Micheletti C., Marenduzzo D., Orlandini E., Summers D. (2006). Knotting of random ring polymers in confined spaces. J. Chem. Phys..

[14] Dai L., van der Maarel J.R.C., Doyle P.S. (2012). Effect of nanoslit confinement on the knotting probability of circular DNA. ACS Macro. Lett..

[15] Dai L., Renner C.B., Doyle P.S. (2015). Metastable knots in confined semiflexible chains. Macromolecules.

[16] Vologodskii A. (2006). Brownian dynamics simulation of knot diffusion along a stretched DNA molecule. Biophys. J..

[17] Huang L., Makarov D.E. (2007). Langevin dynamics simulations of the diffusion of molecular knots in tensioned polymer chains. J. Phys. Chem. A..

[18] Virnau P., Kantor Y., Kardar M. (2005). Knots in globule and coil phases of a model polyethylene. J. Am. Chem. Soc..

[19] D’Adamo G., Micheletti C. (2015). Molecular crowding increases knots abundance in linear polymers. Macromolecules.

[20] Poier P., Likos C.N., Matthews R. (2014). Influence of rigidity and knot complexity on the knotting of confined polymers. Macromolecules.

[21] Matthews R., Louis A.A., Likos C.N. (2012). Effect of bending rigidity on the knotting of a polymer under tension. ACS Macro. Lett..

[22] Rosa A., Di Ventra M., Micheletti C. (2012). Topological jamming of spontaneously knotted polyelectrolyte chains driven through a nanopore. Phys. Rev. Lett..

[23] Narsimhan V., Renner C.B., Doyle P.S. (2016). Translocation dynamics of knotted polymers under a constant or periodic external field. Soft Matter.

[24] Grosberg A.Y., Rabin Y. (2007). Metastable tight knots in a wormlike polymer. Phys. Rev. Lett..

[25] Dai L., Renner C.B., Doyle P.S. (2014). Metastable tight knots in semiflexible chains. Macromolecules.

[26] Dai L., Renner C.B., Doyle P.S. (2015). Origin of metastable knots in single flexible chains. Phys. Rev. Lett..

[27] Grosberg A.Y. (2016). Do knots self-tighten for entropic reasons?. Polym. Sci. Ser. A.

[28] Arai Y., Yasuda R., Akashi K., Harada Y., Miyata H., Kinosita K., Itoh H. (1999). Tying a molecular knot with optical tweezers. Nature.

[29] Bao X.R., Lee H.J., Quake S.R. (2003). Behavior of complex knots in single DNA molecules. Phys. Rev. Lett..

[30] Metzler R., Reisner W., Riehn R., Austin R., Tegenfeldt J.O., Sokolov I.M. (2006). Diffusion mechanisms of localised knots along a polymer. Eur. Phys. Lett..

[31] Tang J., Du N., Doyle P.S. (2011). Compression and self-entanglement of single DNA molecules under uniform electric field. Proc. Natl. Acad. Sci. USA.

[32] Renner C.B., Doyle P.S. (2015). Stretching self-entangled DNA molecules in elongational fields. Soft Matter.

[33] Ercolini E., Valle F., Adamcik J., Witz G., Metzler R., De Los Rios P., Roca J., Dietler G. (2007). Fractal dimension and localization of DNA knots. Phys. Rev. Lett..

[34] Plesa C., Verschueren D., Pud S., van der Torre J., Ruitenberg J.W., Witteveen M.J., Jonsson M.P., Grosberg A.Y., Rabin Y., Dekker C. (2016). Direct observation of DNA knots using a solid-state nanopore. Nat. Nanotechnol..

[35] Dai L., Doyle P.S. (2016). Effects of intrachain interactions on the knot size of a polymer. Macromolecules.

[36] Orlandini E., Baiesi M., Zonta F. (2016). How local flexibility affects knot positioning in ring polymers. Macromolecules.

[37] Plimpton S. (1995). Fast parallel algorithms for short-range molecular dynamics. J. Comput. Phys..

[38] Kremer K., Grest G.S. (1990). Dynamics of entangled linear polymer melts: A molecular-dynamics simulation. J. Chem. Phys..

[39] Frankkamenetskii M.D., Vologodskii A.V. (1981). Topological aspects of polymer physics—Theory and its biophysical applications. Sov. Phys. Usp..

[40] Narsimhan V., Renner C.B., Doyle P.S. (2016). Jamming of knots along a tensioned chain. ACS Macro. Lett..

[41] Murphy M., Rasnik I., Cheng W., Lohman T.M., Ha T. (2004). Probing single-stranded DNA conformational flexibility using fluorescence spectroscopy. Biophys. J..

